# Editorial: Intratympanic and surgical treatment for Meniere's disease

**DOI:** 10.3389/fneur.2022.1072659

**Published:** 2022-12-21

**Authors:** Jun Yang, Yupeng Liu, Maoli Duan

**Affiliations:** ^1^Department of Otorhinolaryngology-Head and Neck Surgery, Xinhua Hospital, Shanghai Jiaotong University School of Medicine, Shanghai, China; ^2^Ear Institute, Shanghai Jiaotong University School of Medicine, Shanghai, China; ^3^Shanghai Key Laboratory of Translational Medicine on Ear and Nose diseases, Shanghai, China; ^4^Ear Nose and Throat Patient Area, Trauma and Reparative Medicine Theme, Karolinska University Hospital, Stockholm, Sweden; ^5^Division of Ear, Nose, and Throat Diseases, Department of Clinical Science, Intervention and Technology, Karolinska Institutet, Stockholm, Sweden

**Keywords:** Ménière's disease, intratympanic, gentamycin, surgery, endolymphatic sac, semicircular canal

## Introduction

The treatment of Meniere's disease (MD) remains controversial and challenging. The issue of how to control vertigo while preserving auditory-vestibular function and improving quality of life is a practical dilemma for both doctors and patients. Intratympanic and surgical treatments are both recommended by the latest Clinical Practice Guideline: Ménière's disease (1) when conservative treatment is ineffective. Intratympanic steroid (ITS) injection and intratympanic gentamicin (ITG) injection are effective treatments for vertigo control in MD patients. ITS has the advantage of protecting hearing, while ITG is more effective for controlling vertigo. ITS is suitable for bilateral disease. The value of endolymphatic sac surgery is that vertigo control and hearing preservation can be achieved concurrently. Furthermore, the risk of endolymphatic sac surgery is low, and serious postoperative complications are rare. Triple semicircular canal occlusion (TSCO) is an alternative procedure for treating MD and is widely accepted by Chinese otolaryngologists. However, the 2020 American guideline (1) and the 2017 European consensus (2) did not recommend TSCO as a selective surgical intervention. Further evidence-based studies and the mechanism of TSCO are needed to achieve global acceptance. The Research Topic “*Intratympanic and Surgical Treatment for Meniere's disease*” consists of seven original articles and two reviews. We put the spotlight on these nine published studies in this Research Topic, as well as the following categories: Pathogenesis Research; Diagnostic Tool; and Intratympanic Treatment and Surgical Treatment.

## Pathogenesis research

The pathogenesis of MD remains unclear. Numerous studies have confirmed that the endolymphatic sac (ES) can be stimulated by antigens and generate an immune response. Immune-related diseases, such as allergies and autoimmune diseases, play an important role in the pathogenesis of MD. The pathogenesis of MD is related to various cytokines and inflammatory mediators of allergies and autoimmune diseases. Huang et al. were the first to successfully directly identify cytokines in the human luminal fluid of the ES when they detected the upregulated expression of TNF-a, IL-6, and IFN-g in the luminal fluid of the ES in MD patients. This modified method provided solid evidence of immune activities in the ES of MD patients. Rizk et al. (3) summarized the published literature on the pathogenesis and etiology of MD from 1917 to 2021 in their latest review. They concluded that there has been a surge in immunologic research on the pathogenesis of MD over the last 20 years (3).

MD patients have a history of abnormal mood, mental anxiety, and overwork before the onset of symptoms. Autonomic nerve dysfunction can increase sympathetic nerve excitability, induce small blood vessel spasms, and raise vascular osmotic pressure. As a result, the microcirculation disturbance of the endolymph leads to the manifestations of MD. Kitahara et al. reported that long-term high levels of vasopressin may adversely affect inner ear microcirculation and endolymph metabolism, leading to the development of MD symptoms. After effective treatment of MD, patients' vasopressin levels can be maintained at low levels. However, there has been little research investigating vasopressin in MD in recent years; therefore, the mechanism of the effect of vasopressin on the inner ear still needs to be clarified.

## Diagnostic tool

Wideband tympanometry (WBT) can sense the lesions of different structures of the middle ear using the probe tone in the frequency range of 0.25–8 kHz, which is better at detecting middle ear function than traditional acoustic immittance. Additionally WBT can indirectly detect inner ear pressure. Therefore, it can be applied to the diagnosis of inner ear diseases. In this Research Topic, an overall summary of the role of WBT in the diagnosis of MD was provided by Meng et al. They found differences between MD patients and normal individuals in terms of resonance frequency, absorbance, integral area of absorbance, and G-Width. The application of WBT in MD is based on the hypothesis that pressure changes caused by endolymphatic hydrops (EH) alter the tension of the annular ligament and the “third window” of the inner ear, and in the case of an intact ossicular chain, decreased mobility of the stapedial footplate results in the reduction of tympanic membrane compliance. At present, a broad range of clinical applications of WBT is not possible due to the small sample sizes in studies, a lack of follow-up results, and a lack of data in different age populations and ethnic groups. WBT can be altered as part of a test battery if further evidence-based studies in the field are improved.

## Intratympanic gentamycin

Aminoglycosides are ototoxic to both vestibular and cochlear hair cells. Gentamicin is the first preference for intratympanic injection due to its greater vestibular toxicity compared with cochlear toxicity. ITG is well tolerated, controls vertigo attacks, and has a low incidence of severe hearing loss. The 2020 Clinical Practice Guideline (1) recommended administering ITG to patients with unilaterally active MD who are refractory to conservative non-destructive treatments. Caution must be exercised in patients with bilateral MD because ITG carries the risk of significant bilateral vestibular hypofunction and hearing loss. Wegmann-Vicuña et al. evaluated the effect of ITG and concluded that 59.6% of the patients in their sample group achieved complete vertigo control after one single dose of ITG with a follow-up period of up to 6 years. Patients receiving a single ITG injection showed less frequent chronic disequilibrium and demand of vestibular rehabilitation. They also suggested that the endpoint of treatment was a vestibulo-ocular reflex (VOR) gain reduction of >17.8%. The generally accepted treatment endpoint of ITG is when the patient does not have a vertigo attack over a 12-month period or a vestibular loss in objective tests in the affected ear (4). Guan et al. completed a retrospective study on the effectiveness of ITG treatment and the value of the Halmagyi head thrust test (HTT) for predicting treatment durability. It is noteworthy that the authors used traditional HTT instead of a video Head Impulse Test (vHIT) to identify the impact of vestibular function. The HTT can be performed without specialized equipment and is a facile test that can be performed anywhere. Clinicians should never forget to use it when resources are limited.

## Surgical treatment

Non-ablative surgery is a dispensable component of surgical treatment for MD patients with usable hearing and residual vestibular function when conservative treatment is ineffective. Histopathological change of EH has inspired surgeons to manipulate the membranous labyrinth for the purpose of relieving vertigo and preserving hearing. Kersbergen and Ward reviewed the history of manipulation of the membranous labyrinth. Femenic shunt, Fick sacculotomy, tack operation, cochleosacculotomy, otic-perotic shunt, and semicircular canal plugging were respectively introduced. The aim of these operations, with the exception of canal plugging, is to relieve vertigo and improve hearing by draining excess endolymph. The authors summarized the published literature on the outcomes of vertigo control and hearing function. Vertigo control was satisfactory in most cases, but hearing function was not well preserved. Although these procedures have been abandoned by clinicians, they remain as great innovations in the history of otologic surgery. Jiang et al. investigated the surgical outcomes of TSCO and vestibular nerve resection. Vertigo control rate was satisfactory in both groups; however, a lower rate of postoperative paroxysmal dizziness and unsteadiness was observed in the TSCO group. The limitation of TSCO is the small number of large studies associated with it, which were carried out by Chinese clinicians (5, 6). Further research on the mechanism of TSCO and evidence-based studies are required.

Endolymphatic sac surgery was not recommended by the 2020 American guideline due to uncertainty about its benefits and its discordant underlying mechanisms. Zhang et al. reported a series of cases of patients who underwent resection of the lateral wall of the endolymphatic sac. Complete vertigo control was achieved in 61.6% of the patients. The authors speculated that the procedure's mechanism involved the reduction of endolymphatic fluid secretions and the elimination of the immune response of the endolymphatic sac. Zheng et al. compared the surgical effect of local endolymphatic sac decompression, endolymphatic mastoid shunt, and wide endolymphatic sac decompression. The authors concluded that wide endolymphatic sac decompression has a higher vertigo control rate (88.6%), better improvement of QOL, and relatively higher hearing stability. Paparella first proposed wide endolymphatic sac decompression and speculated that this procedure could enhance the blood supply of the sac and improve its absorption function (7). It is interesting that the two seemingly discordant surgical approaches achieved similar outcomes. The role of the endolymphatic sac in EH and MD remains a mystery.

## Conclusions

In summary, the nine articles presented in the Research Topic “Intratympanic and Surgical Treatment for Meniere's disease” may provide more information about the field. Challenges in diagnosing and treating MD patients still remain. First, MD is often accompanied by other diseases. Patients with long-term recurrent attacks are likely to have psychological disorders or mental illness. Second, owing to the long-term nature and repetitiveness of MD, the economic costs of diagnosis, treatment, and rehabilitation are notable. Third, the factors that affect the treatment outcomes of MD are complex, and therefore, further studies are needed.

## Author contributions

All authors listed have made a substantial, direct, and intellectual contribution to the work and approved it for publication.
